# Electrochemical Detection of Alzheimer's Disease Biomarkers using Graphene based Biosensors

**DOI:** 10.1002/alz70856_098540

**Published:** 2025-12-24

**Authors:** Sophia Nazir, Yinghui Wei, Genhua Pan

**Affiliations:** ^1^ University of Plymouth, Plymouth, Plymouth, United Kingdom; ^2^ University of Plymouth, Plymouth, United Kingdom

## Abstract

**Background:**

Alzheimer's disease (AD) is a leading cause of neurodegenerative dementia, affecting over 50 million people worldwide. Early‐stage detection of AD is critical, as no disease‐modifying treatments or preventive strategies are currently available. However, the lack of sensitive and minimally invasive diagnostic tools for early AD detection remains a significant barrier to effective clinical trials, often contributing to trial failures.

**Method:**

In this study, we report on the development of an ultrasensitive electrochemical immunosensor for the detection of AD biomarkers, specifically tau‐441 and tau‐217, in body fluids. The sensor utilizes a graphene‐based electrochemical platform, which was fabricated as part of the EU Horizon Europe MSCA‐ITN‐DN CombiDiag project. The immunosensor operates through a printed graphene biosensor designed for the detection of body fluid biomarkers in the context of early‐stage dementia.

**Result:**

The proposed immunosensor demonstrates an impressive detection range from 1 fM to 1 pM with a femtomolar‐level limit of detection (LoD), which is the lowest reported LoD for a graphene‐based biosensor. This enhanced sensitivity is attributed to the use of a 3D graphene foam material, which offers a large surface area, increased reproducibility, and numerous binding sites for antibodies, leading to improved sensor performance.

**Conclusion:**

The high sensitivity of the developed biosensor enables the detection of AD biomarkers at low concentrations in various body fluids. This makes it applicable for monitoring biomarkers in different stages of disease progression and could be a valuable tool for early‐stage diagnosis and inclusion in clinical drug trials for AD.

